# Melatonin Alleviates Neuroinflammation and Metabolic Disorder in DSS-Induced Depression Rats

**DOI:** 10.1155/2020/1241894

**Published:** 2020-07-30

**Authors:** Wei-jie Lv, Cui Liu, Lin-zeng Yu, Jia-hao Zhou, Yue Li, Ying Xiong, Ao Guo, Li-min Chao, Qian Qu, Guang-wei Wei, Xing-gang Tang, Yu-long Yin, Shi-ning Guo

**Affiliations:** ^1^Guangdong Laboratory for Lingnan Modern Agriculture/College of Veterinary Medicine, South China Agricultural University/Guangdong Technology Research for Traditional Chinese Veterinary Medicine and Natural Medicine, Guangzhou 510642, China; ^2^Institute of Animal Health, Guangdong Academy of Agricultural Sciences, Guangzhou 510640, China; ^3^College of Animal Medicine, South China Agricultural University, Guangzhou 510642, China; ^4^Key Laboratory of Agro-Ecological Processes in Subtropical Region, Institute of Subtropical Agriculture, Chinese Academy of Sciences, Hunan Provincial Key Laboratory of Animal Nutritional Physiology and Metabolic Process, Changsha, China

## Abstract

There is a bidirectional relationship between inflammatory bowel disease (IBD) and depression/anxiety. Emerging evidences indicate that the liver may be involved in microbiota-gut-brain axis. This experiment focused on the role of melatonin in regulating the gut microbiota and explores its mechanism on dextran sulphate sodium- (DSS-) induced neuroinflammation and liver injury. Long-term DSS-treatment increased lipopolysaccharide (LPS), proinflammation cytokines IL-1*β* and TNF-*α*, and gut leak in rats, breaking blood-brain barrier and overactivated astrocytes and microglia. Ultimately, the rats showed depression-like behavior, including reduction of sucrose preference and central time in open field test and elevation of immobility time in a forced swimming test. Oral administration with melatonin alleviated neuroinflammation and depression-like behaviors. However, melatonin supplementation did not decrease the level of LPS but increase short-chain fatty acid (SCFA) production to protect DSS-induced neuroinflammation. Additionally, western blotting analysis suggested that signaling pathways farnesoid X receptor-fibroblast growth factor 15 (FXR-FGF 15) in gut and apoptosis signal-regulating kinase 1 (ASK1) in the liver overactivated in DSS-treated rats, indicating liver metabolic disorder. Supplementation with melatonin markedly inhibited the activation of these two signaling pathways and its downstream p38. As for the gut microbiota, we found that immune response- and SCFA production-related microbiota, like *Lactobacillus* and *Clostridium* significantly increased, while bile salt hydrolase activity-related microbiota, like *Streptococcus* and *Enterococcus*, significantly decreased after melatonin supplementation. These altered microbiota were consistent with the alleviation of neuroinflammation and metabolic disorder. Taken together, our findings suggest melatonin contributes to reshape gut microbiota and improves inflammatory processes in the hippocampus (HPC) and metabolic disorders in the liver of DSS rats.

## 1. Introduction

Inflammatory bowel disease (IBD), including Crohn's disease and ulcerative colitis, is a chronic inflammatory disease that negatively affects the life quality of patients. When compared with healthy individuals, these diseases are clearly associated with mental dysfunction [1, 2]. More than 30% of IBD patients were accompanied with mental disorders, including anxiety and depression [3]. Chronic diseases can lead to psychological disorders along with interpersonal relationships, family, work, and social stress [4, 5]. At the same time, sustained stress may induce physical dysfunction, which in turn leads to immunosuppression, gut permeability, and other inflammation changes that may eventually result in chronic diseases, including IBD [6, 7]. In recent years, more and more researches have focused on the relationship between mental disease and intestinal inflammation. We speculate that the mechanism may be caused by a disorder of the microbiota-gut-brain axis. Through longitudinal follow-up trials, they found that mental disease may be associated with poor prognosis of IBD and that intestinal inflammatory activity is also related to the development of mental disorders [8–10]. Additionally, there is a bidirectional relationship between IBD and mental disorders [11]. These bidirectional brain-gut pathways have been reported to play a key role in functional gastrointestinal disorders such as IBS and functional dyspepsia [12, 13]. Stimulation of stress, anxiety, and depression can increase the burden on patients with IBD [14], although there is still controversy between stress and IBD [15].

Data from recent years indicate that the gut microbiome is closely related to the function of the liver system [16, 17]. Alteration in gut microbiome has been reported in patients with various liver disorders including fibrosis and cancer and has been validated in animal disease models [18–21]. The liver affects the structure of the gut microbiota by regulating the *Clostridium*-mediated bile acid production [22, 23]. In turn, the gut microbiome can influence the liver function through reabsorption in the terminal ileum [24, 25]. Recent researches suggest that liver diseases were associated with depression and suicide attempts [26]. Germ-free mice underwent fecal microbiota transplantation from major depressive disorder (MDD) patients resulting in liver metabolic disorder and mainly focusing on three major disturbances, including lipid, amino acid, and energy metabolism [27], which is consistent with our previous study [28]. Depression is often accompanied by insomnia symptoms, and melatonin plays a key role in maintaining circadian rhythm [29, 30]. Recent researches have revealed that melatonin involved in mental control [31], weanling stress [32], sleep deprivation [33], obesity [34], and lipid metabolism [35]. Bile contain melatonin has been reported to improve intestinal epithelial injury [36] and change the structure of gut microbiota, including elevating richness and diversity of microbiota [37], such as *Akkermansia*, *Bacteroides*, and *Faecalibacterium* [33]. At present, IBD and depression have established a preliminary link through gut microbiota [1–3]. Also, previous studies have shown metabolic disorders in the liver of MDD and depressive rats [27, 28]. Although melatonin has a certain effect in the treatment of depression, its effect on liver damage and neuroinflammation caused by depression is not yet clear.

To address this issue, rats were treated with DSS to evaluate whether chronic colitis was linked to the development of depression/anxiety and liver metabolic disorder. Western blotting analyses indicate that melatonin reversed dysmetabolism. By sequencing the 16S rRNA gene of rat feces, we found that *Lactobacillus* and *Clostridium*, immune-modulating, SCFA, and bile acid production microbiota may serve as a potential mechanism.

## 2. Materials and Methods

### 2.1. Animal Treatment

Adult male SPF SD rats (6 weeks), each weighing 200 ± 20 g, were purchased from the Experimental Animal Center of Southern Medical University (Guangzhou, China). Rats were randomly divided into three groups: (1) CON group—rats were fed a standard diet; (2) DSS group—rats were treated with 1.5% DSS for induction of colitis model as previous described [38]. For induction of chronic DSS colitis, each rat received 4 cycles of DSS treatment consisting of 7 days with 1.5% DSS in the drinking water followed by a 10-day recovery phase with normal drinking water. After the last DSS cycle, rats received normal drinking water for 2 weeks. (3) The third group is the melatonin group (MT) wherein DSS rats were fed with melatonin (100 mg/Kg) by gavage at 7:00 AM for 2 weeks. The dose was chosen based on previous researches with minor modification [37, 39]. The study was approved by the Southern Medical University Experimental Animal Ethics Committee. All experimental procedures were performed in accordance with the relevant guidelines approved by the Experimental Animal Ethics Committee of Southern Medical University.

### 2.2. Behavioral Testing

#### 2.2.1. Sucrose Preference Test (SPT)

The test was performed on the 28th day as previously described [40]. After 24 hours of water ban, each rat was placed in a single cage and two bottles containing water and 1% sucrose solution were placed. The ratio of the consumption of the sucrose solution to the amount of total solution consumed in one hour represents a parameter of the pleasure behavior.

#### 2.2.2. Open Field Test (OFT)

The test was performed as previously described [41]. Briefly, all rats were individually tested in a device consisting of a black square substrate (50∗50 cm) and a black wall (50 cm). Rats were placed in the corners of the device, and after 1 minute of adaptation, the rats were free to move for 5 minutes using a video-computerized tracking system. The total activity time is used as an indicator of activity, and the time spent in the central area (36% of surface area) is used as an indicator of depression behavior.

#### 2.2.3. Forced Swimming Test (FST)

Rats were placed in a cylinder (30 cm × 45 cm) filled with water at a temperature of 25°C, for a 6-minute period. The duration of immobility in seconds was monitored during the last 4 min of the 6 min test. The immobility period was defined as the time spent by the animal floating in the water without struggling and making only movements necessary to keep its head above the water. Immediately afterwards, the trial rats were placed under a heating lamp to dry [42].

### 2.3. Sample Collection

Anesthesia was performed with sodium pentobarbital. Serum was collected from the abdominal aorta and centrifuged at 3500 rpm for 3 minutes at 4°C. Fecal stools, the liver, the colon, and the hippocampus were collected and frozen in liquid nitrogen and maintained at -80°C for detection. The colon and liver samples were collected and fixed in 3.7% formalin for detection.

### 2.4. 16S rRNA Gene Sequence Analysis

Total bacterial genomic DNA samples were extracted using the Fast DNA SPIN extraction kits (MP Biomedicals, Santa Ana, CA, USA). The quantity and quality of extracted DNAs were measured using a NanoDrop ND-1000 spectrophotometer (Thermo Fisher Scientific, Waltham, MA, USA) and agarose gel electrophoresis, respectively. PCR amplification of the bacterial 16S rRNA genes (V4–V5 region) was performed using the forward primer 515F (5′-GTGCCAGCMGCCGCGGTAA-3′) and the reverse primer 907R (5′-CCGTCAATTCMELATONINTTRAGTTT-3′).

### 2.5. Sequence Analysis

The Quantitative Insights Into Microbial Ecology (QIIME, v1.8.0) pipeline was employed to process the sequencing data, as previously described [43]. Briefly, raw sequencing reads with exact matches to the barcodes were assigned to respective samples and identified as valid sequences. The low-quality sequences were filtered through the following criteria [44, 45]: sequences that had a length of <150 bp, sequences that had average Phred scores of <20, sequences that contained ambiguous bases, and sequences that contained mononucleotide repeats of >8 bp. Paired-end reads were assembled using FLASH [46]. After chimera detection, the remaining high-quality sequences were clustered into operational taxonomic units (OTUs) at 97% sequence identity by UCLUST [47]. A representative sequence was selected from each OTU using default parameters. OTU taxonomic classification was conducted by BLAST searching the representative sequence set against the Greengenes database [48] using the best hit [49]. An OTU table was further generated to record the abundance of each OTU in each sample and the taxonomy of these OTUs. OTUs containing less than 0.001% of total sequences across all samples were discarded. To minimize the difference of sequencing depth across samples, an averaged, rounded rarefied OTU table was generated by averaging 100 evenly resampled OTU subsets under the 90% of the minimum sequencing depth for further analysis.

### 2.6. Bioinformatics and Statistical Analysis

Sequence data analyses were mainly performed using QIIME and R packages (v3.2.0). OTU-level alpha diversity indices, such as Chao1 richness estimator, ACE metric (Abundance-based Coverage Estimator), Shannon diversity index, and Simpson index, were calculated using the OTU table in QIIME. OTU-level ranked abundance curves were generated to compare the richness and evenness of OTUs among samples. Beta diversity analysis was performed to investigate the structural variation of microbial communities across samples using UniFrac distance metrics [50, 51] and visualized via principal coordinate analysis (PCoA), nonmetric multidimensional scaling (NMDS), and unweighted pair-group method with arithmetic mean (UPGMA) hierarchical clustering [52]. Differences in the UniFrac distances for pairwise comparisons among groups were determined using Student's *t*-test and the Monte Carlo permutation test with 1000 permutations and visualized through the box-and-whisker plots. Principal component analysis (PCA) was also conducted based on the genus-level compositional profiles [52]. The significance of differentiation of microbiota structure among groups was assessed by PERMANOVA (permutational multivariate analysis of variance) and ANOSIM (analysis of similarities) [53] using R package “vegan.” The taxonomy compositions and abundances were visualized using MEGAN [54] and GraPhlAn [55]. Venn diagram was generated to visualize the shared and unique OTUs among samples or groups using R package “VennDiagram,” based on the occurrence of OTUs across samples/groups regardless of their relative abundance [56]. Taxa abundances at the phylum, class, order, family, genus, and species levels were statistically compared among samples or groups by Metastats [57] and visualized as violin plots. LEfSe (linear discriminant analysis effect size) was performed to detect differentially abundant taxa across groups using the default parameters [58]. PLS-DA (partial least squares discriminant analysis) was also introduced as a supervised model to reveal the microbiota variation among groups, using the “plsda” function in R package “mixOmics” [59]. Random forest analysis was applied to discriminating the samples from different groups using the R package “randomForest” with 1,000 trees and all default settings [60, 61]. The generalization error was estimated using 10-fold cross-validation. The expected “baseline” error was also included, which was obtained by a classifier that simply predicts the most common category label. Co-occurrence analysis was performed by calculating Spearman's rank correlations between predominant taxa. Correlations with ∣RHO | >0.6 and *p* < 0.01 were visualized as co-occurrence network using Cytoscape [62]. Microbial functions were predicted by PICRUSt (phylogenetic investigation of communities by reconstruction of unobserved states), based on high-quality sequences [63].

### 2.7. Short-Chain Fatty Acid Analysis

Fecal samples were collected using an Agilent 7890A/5975C gas chromatograph (Agilent Technologies, Inc., Palo Alto) to determine short-chain fatty acids (SCFA; acetic acid and propionic acid) according to a previous study [64].

### 2.8. Western Blot

Total proteins from liver and colon samples were extracted using protein extraction reagents (Thermo Fisher Scientific, Waltham, MA, USA), and 30 *μ*g proteins were separated by a reducing SDS-PAGE electrophoresis. Then, the proteins were transferred onto a PVDF membrane (Millipore, Billerica, MA, USA) and blocked with 5% nonfat milk in Tris-Tween-buffered saline buffer for 1.5 hours. Then, the membranes were incubated with primary antibodies and then incubated with horseradish peroxidase-conjugated secondary antibodies. The gray values of the bands were calculated using ImageJ software and were normalized to actin. 1 : 500 for rabbit anti-FXR (Abcam, Cambridge, MA), 1 : 1000 for mouse anti-FGF15 (Santa Cruz Biotechnology, USA), ASK1 (28201-1-AP, 1 : 750, Proteintech), p-ASK1 (#3764, 1 : 1000, CST), p38 (#9212, 1 : 1000, CST), p-p38 (9211 s, 1 : 1000, CST), and *β*-actin (66009-1-Ig, 1 : 5000, Proteintech).

### 2.9. Quantitative Image Analysis

Immunofluorescence was performed as previously described [65], with the following modification: primary antibody—rabbit anti-GFAP (1 : 2000; Ab5076/Ab10062, Abcam, UK) 8 h at 4°C. To detect primary antibodies, a suitable secondary antibody conjugated to FITC-conjugated donkey anti-mouse IgG (1 : 400, A21202, Life technologies, USA) was used. The sample was covered with a mounting medium (S2100, Solarbio, China) and observed with an epifluorescence microscope (DM1000, Leica, German).

### 2.10. Pathological Changes of the Colon and Liver

The colon and liver were collected following animal sacrifice. Subsections were partially embedded in 3.7% formalin solution (Sigma, USA). Paraffin-embedded colon sections (4-5 *μ*m) were stained with hematoxylin and eosin (H&E) for morphological examination, then observed with an Olympus BH22 microscope (Japan).

### 2.11. Proinflammatory Cytokines and Markers of Intestinal and Blood-Brain Barrier Analysis

Inflammatory cytokines such as IL-1*β* and zonulin in the hippocampus; zonulin, IL-1*β*, and TNF-*α* in the colon; and LPS in the plasma were tested using an ELISA kit (Cusabio, Houston, TX, USA; https://www.cusabio.com/).

### 2.12. Statistical Analysis

Statistical analyses were approached using SPSS version 22 (SPSS, Inc., Chicago, IL, USA) and GraphPad Prism 5. The results such as *α*-diversity, behavior data, IL-1*β*, TNF-*α*, LPS, zonulin, histology score, activated cells in the hippocampus, and protein were presented as the mean ± SEM. Data sets were assessed by one-way analysis of variance (ANOVA) followed by Bonferroni's post hoc test.

## 3. Results

### 3.1. Melatonin Reduces DSS-Induced Depression/Anxiety-Like Behavior

**Figure 1:**
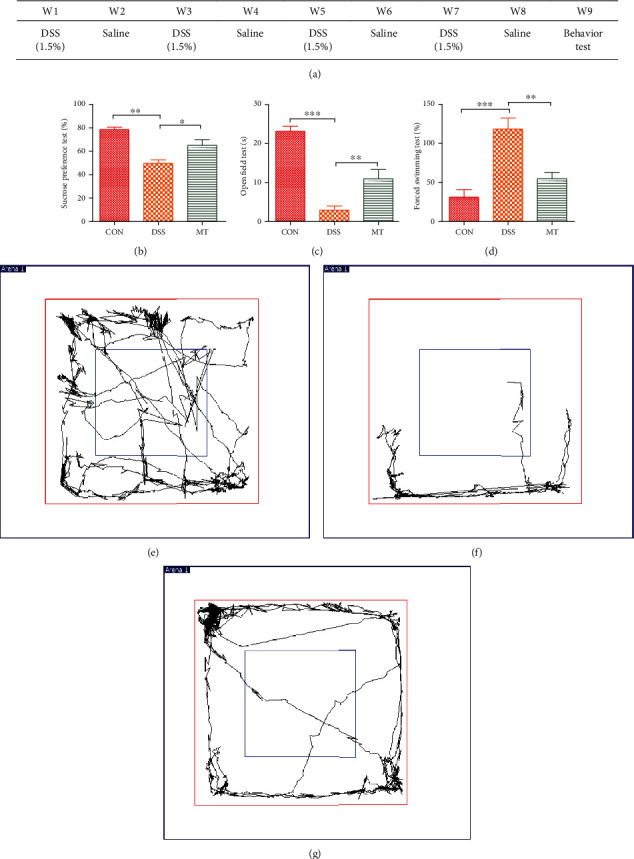
Behavior tests after rats were treated with DSS and melatonin. (a) The development of depression induced by DSS. (b) Results of sucrose preference test. (c) Results of open field test. (d) Results of forced swimming test. (e–g) Representative motion tracks for the CON group, the DSS group, and the MT group. Data represent the mean ± SEM. ^∗^*p* < 0.05; ^∗∗^*p* < 0.01. CON = 7; DSS = 5; MT = 6.

Since previous studies have reported that patients with inflammatory bowel disease are often accompanied with mental health issues [66], we evaluate the behavior tests after rats were treated with DSS. In this study, chronic colitis rats were used to verify whether DSS-induced IBD promotes the development of depression/anxiety behavior (Figure 1(a)). In a sucrose preference test, sucrose preference of DSS rats (DSS) was decreased significantly compared with control group (CON) rats (Figure 1(b)). In an open field test, the central area time of DSS rats was significantly reduced compared with control rats (Figure 1(c)). In a forced swimming test, immobile durations of DSS rats were significantly increased compared with control rats (Figure 1(d)). Additionally, the motion tracks of CON rat and DSS rat in the open field test are shown in Figures 1(e) and 1(f). By contrast, supplementation with melatonin reversed these changes (Figures 1(b), 1(c), 1(d), and 1(g)).

### 3.2. Melatonin Reprograms Gut Microbiota in DSS-Treated Rats

#### 3.2.1. *α* Diversity

**Figure 2:**
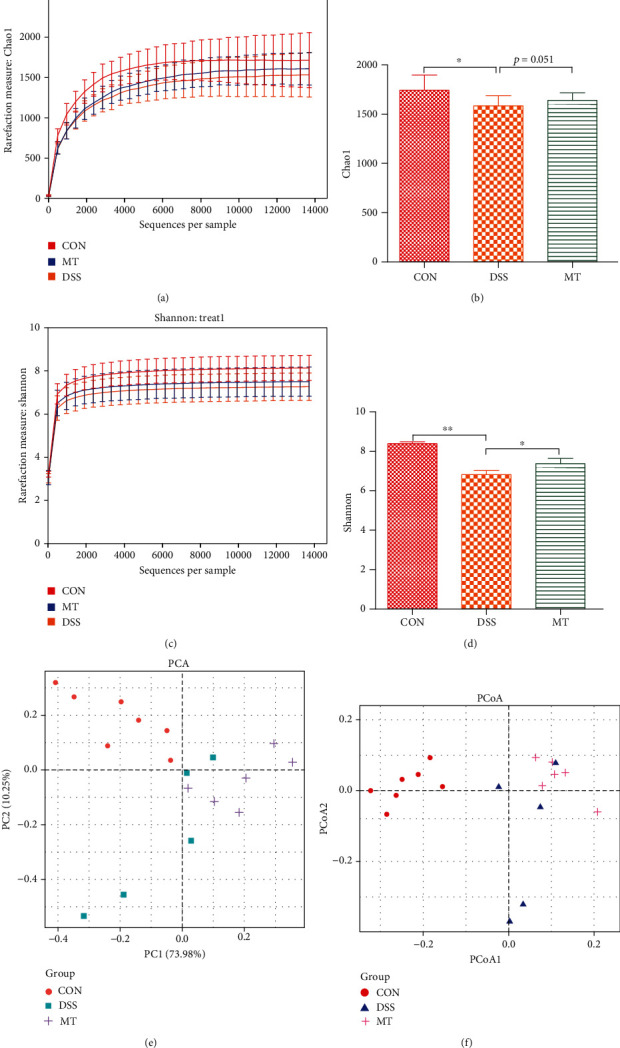
Melatonin altered gut microbiota structure in DSS rats. (a, b) Chao1 index analysis. (c, d) Shannon index analysis. (e, f) PCA and PCoA plot analysis. Data represent the mean ± SEM. ^∗^*p* < 0.05; ^∗∗^*p* < 0.01. CON = 7; DSS = 5; MT = 6.

Previous studies have reported that depression can be alleviated via the alteration of gut microbiome [67, 68]. Chao1 is an index to estimate the number of OTUs in the community using the Chao1 algorithm. Chao1 is commonly used in ecology to estimate the total number of species. The Shannon diversity index (or Shannon-Wiener index) is a diversity index that is commonly used to characterize species diversity in a community. It is a measure of the species diversity of an ecosystem based on information theory. In our study, *α* diversity, as measured by the Chao1 and Shannon indices, was significantly reduced after treatment with DSS, which means the richness and diversity of species decreased. Interestingly, treatment with melatonin markedly increased Chao1 and Shannon indexes, suggesting an improvement in gut microbiota richness and diversity in DSS-treated rats (Figures 2(a)–2(d)).

#### 3.2.2. *β* Diversity

Based on the unweighted UniFrac distance calculation, CON and DSS rats presented a distinct clustering of microbiota community structure (Figures 2(e) and 2(f)). Obviously, we can observe the difference in the gut microbiota between different groups through the distance between the samples. The longer the distance between CON and DSS/MT, the greater the difference in gut microbiota; on the contrary, the closer the distance between DSS and MT samples, the smaller the difference in gut microbiota between them; however, MT has a tendency to separate from DSS. Although the microbial community structure of DSS and melatonin rats was not completely separated, the diversity of melatonin has a distinct trend. These results suggest that the intestinal microbiota structure of rats is changed after DSS and melatonin intervention.

**Figure 3:**
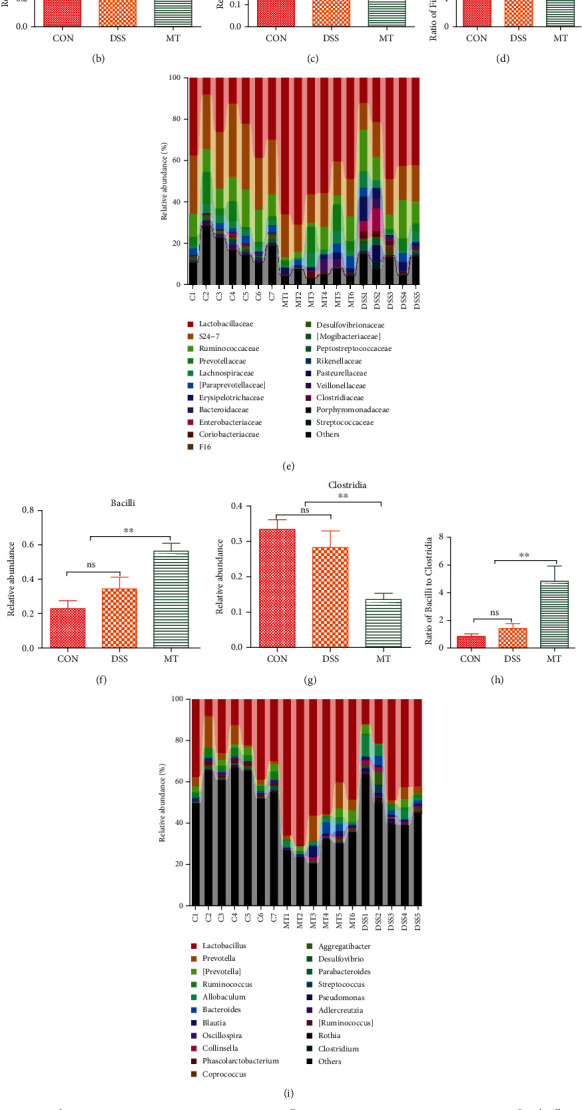
Melatonin improved gut microbiota in DSS rats. (a–d) Microbiota compositions in phylum level. (e–h) Microbiota compositions in family level. (i–m) Microbiota compositions in genera level. (n, o) SCFAs change when rats were treated with DSS and melatonin. Data represent the mean ± SEM. ^∗^*p* < 0.05; ^∗∗^*p* < 0.05. CON = 7; DSS = 5; MT = 6.

To identify the significant changes in the gut microbiota among the three groups, we used QIIME software to obtain the composition and abundance distribution table of each sample at the five classification levels (the phylum, the class, the order, the family, and the genus), and the results of the analysis were presented in a histogram. Here, at the phyla level, the lower ratio of *Firmicutes* to *Bacteroidetes* (*F*/*B*) is considered to be a key index for a healthy state of the gut microbiome [69, 70]. In our study, the ratio of *F*/*B* was decreased in DSS rats and increased in melatonin rats (Figures 3(a)–3(d)). At the class level (Figure 3(e)), there were significant increase in *Bacilli* and significant decrease in *Clostridia* after melatonin treatment (Figures 3(f) and 3(g)), Since *Bacilli* to *Clostridia* (*B*/*C*) has been reported as a novel index of stress effects [71]. So, we measured the ratio of *Bacilli* to *Clostridia*. Here, the ratio of *B*/*C* was higher in MT rats relative to DSS rats (Figure 3(h)). At the genus level (Figure 3(i)), relative abundance of *Streptococcus* and *Enterococcus* is increased in DSS rats and the abolition of the effect by treatment with melatonin (Figures 3(j) and 3(k)). On the other hand, relative abundance of *Lactobacillus* and *Clostridium* is increased by treated with melatonin (Figures 3(l) and 3(m)). Altered microbiota usually change the production of SCFA [34, 72]; in the present study, supplementation with melatonin reversed the reduction of SCFA (acetic acid, propionic acid) induced by treated with DSS (Figures 3(n) and 3(o)). PICRUSt analyses predicted that rats treated with DSS produce more LPS and supplementation with melatonin had no effect to improve this change (Figure S1).

### 3.3. Melatonin Protects against DSS-Induced Inflammation in the Brain and Colon

**Figure 4:**
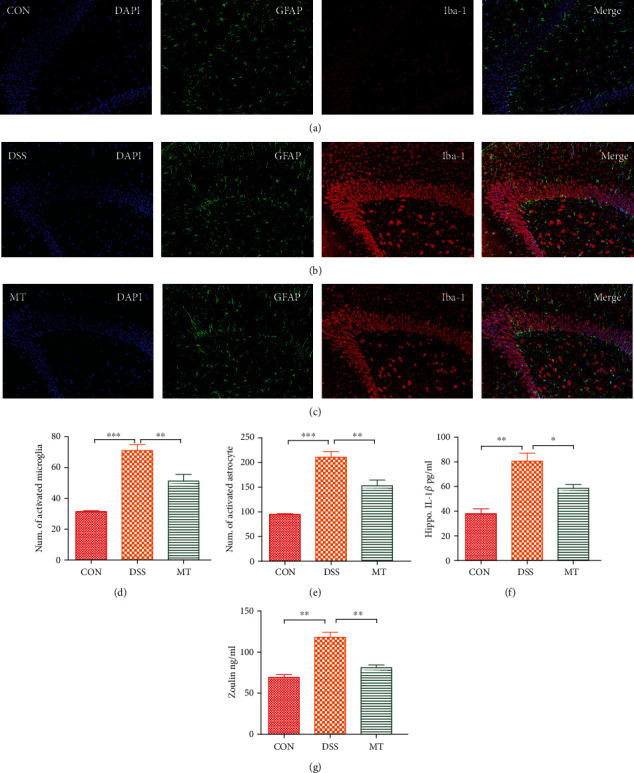
Melatonin-induced attenuation of hippocampal neuroinflammation in DSS rats. (a–c) Micrographs depict labeling of GFAP (green) and Iba-1 (red) in rat hippocampal slices. Nuclear staining was performed with DAPI (blue). (d) The number of cells expressing GFAP, a marker of astrocyte activation. (e) The number of cells expressing Iba1, a marker of microglia activation. (f) Hippocampal IL-1*β* levels of rats measured with ELISA. (g) Hippocampal zonulin levels of rats measured with ELISA. Data represent the mean ± SEM. ^∗^*p* < 0.05, ^∗∗^*p* < 0.01, and ^∗∗∗^*p* < 0.001. CON = 7; DSS = 5; MT = 6.

**Figure 5:**
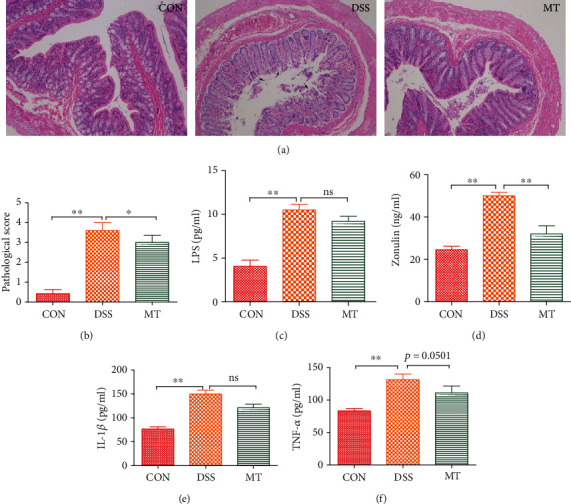
Melatonin altered intestinal morphology and promoted the gut leak in DSS rats. (a) Melatonin influenced intestinal morphology. (b) The histology score of DSS and MT rats. (c) Melatonin had no significant effect to prevent LPS production in the plasma. (d–f) Effects of melatonin in regulating zonulin, IL-1*β*, and TNF-*α* in the colon. Data represent the mean ± SEM. ^∗^*p* < 0.05; ^∗∗^*p* < 0.01. CON = 7; DSS = 5; MT = 6.

Given the evidence that depression can be related to gut and brain inflammatory [42, 71], here, we measured the activities of brain glia astrocytes, microglia, and inflammatory cytokines in the hippocampus. In this research, immunofluorescence was used to analyze astrocytes and microglia. The morphological analysis of GFAP-positive and Iba-1-positive cells revealed that DSS exposure caused an increase in the number of activated astrocytes and microglia in the hippocampus compared to CON rats; furthermore, melatonin treatment induced large reductions in the numbers of activated microglia and astrocytes (Figures 4(a)–4(e)). The effects on microglia and astrocyte activation states were paralleled by alterations in hippocampal IL-1*β* and the blood-brain barrier (BBB) permeability marker zonulin. Melatonin abolished DSS-induced, aberrant increases of both IL-1*β* and zonulin (Figures 4(f) and 4(g)). A recent study showed that melatonin can improve intestinal morphology [32]. In this study, the results of colon pathology of different treatment are shown in Figures 5(a) and 5(b). CON group rats exhibited healthy pathological characteristic, whereas inflammatory cell infiltration of colonic mucosa, degeneration of intestinal villus epithelial cells, necrosis, and shedding were observed in the DSS rats. Melatonin supplementation had a significant effect on intestinal repair, including decreasing the inflammatory cell infiltration of colonic mucosa. Anxiety and depression are associated with imbalanced gut microbiome that secretes LPS endotoxin into plasma, which is correlated with altered integrity of intestinal epithelial cells named zonulin [73]. After rats were treated with DSS, LPS in the plasma and zonulin, IL-1*β*, and TNF-*α* in the colon were elevated significantly. Consistent with the above result, melatonin supplementation had markedly decreased level of zonulin and IL-1*β* except LPS and TNF-*α* in the colon (Figures 5(c)–5(f)).

### 3.4. Melatonin Inhibits the DSS-Induced Activation of FXR and ASK1 Signaling Pathways

**Figure 6:**
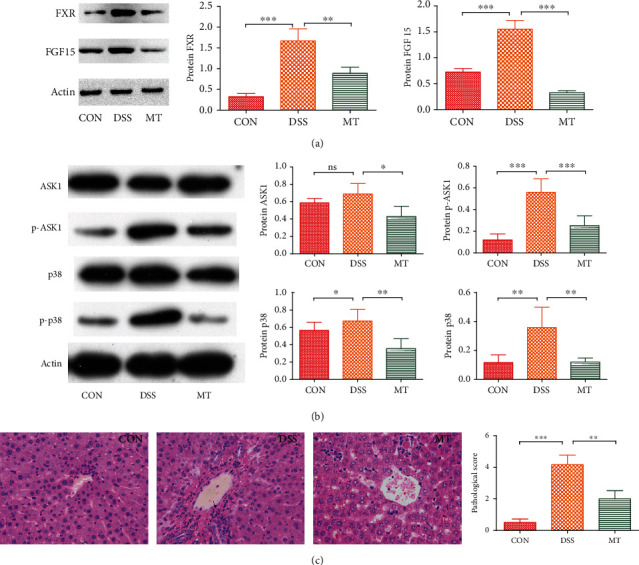
Inhibition of melatonin on FXR-FGF15 and ASK1 signaling pathways in DSS rats. (a) Melatonin inhibited FXR-FGF15 signaling pathways. (b) Total ASK1, phospho-ASK1, total p38, and phospho-p38 protein levels in liver tissue were determined using immunoblotting. (c) HE staining and histology analysis of liver tissue in DSS and melatonin treatment. Data represent the mean ± SEM. ^∗^*p* < 0.05, ^∗∗^*p* < 0.01, and ^∗∗∗^*p* < 0.001. CON = 7; DSS = 5; MT = 6.

A previous study provides the evidence that melatonin alleviates liver metabolic disorder caused by NAFLD via inhibiting ASK1 pathway activation in a *β*-arrestin-1-dependent manner [35]. Huang et al. reported that decreased intestinal bile-salt hydrolase (BSH) microbes and/or decreased FXR-FGF15 signaling may be potential mechanisms behind the cholesterol and lipid lowering [23]. Moreover, our previous research has reported that depression may cause liver lipid metabolic disorder [28]. In this case, we evaluated the influences of melatonin on the FXR and ASK1 signaling pathway in rats with DSS, which have demonstrated correlation with metabolic disorder [23, 34, 35]. The FXR and phosphorylation of ASK1 in the liver tissue of DSS rats were remarkably increased, suggesting that the FXR and ASK1 signaling pathway is activated upon DSS (Figures 6(a) and 6(b)). Relative protein levels of FXR and FGF15 in the colon were decreased in MT rats relative to DSS rats (Figure 6(a)). Melatonin treatment not only suppressed the phospho-ASK1 level but also substantially inhibited the total ASK1 level (Figure 6(b)). We next determined the status of p38, the downstream cascade of ASK1. DSS affected total p38 protein levels and significantly increased the phosphorylation of p38. Conversely, melatonin inhibited the enhanced phosphorylation of p38 (Figure 6(b)). Additionally, the liver histological changes showed that coagulative necrosis caused by DSS can be reversed by melatonin supplementation (Figure 6(c)).

## 4. Discussion

Previous researches have showed that melatonin has a beneficial effect on gut, brain, and liver function, such as alleviating cognition impairment by antagonizing brain insulin resistance in aged rats fed a high-fat diet [31], enhancing neural stem cell differentiation and engraftment [74], protecting against lipid-induced mitochondrial dysfunction [75], and alleviating cadmium-induced liver injury [76]. These effects were achieved by varieties of molecular pathways including differentiation, oxidative stress, immune function, and apoptosis [77–79]. In addition to playing an independent role in the gastrointestinal disorder, the intestinal microbiota also affected many biological functions of other organs and are related to the pathogenesis of various organs [80–82].

Microbiome-gut-brain interactions affect mental health. The presence of anxiety or depression is linked to the development of gastrointestinal symptoms. In turn, the presence of gastrointestinal symptoms is also associated with the development of mental illnesses [83]. Although previous studies have reported that melatonin affects the gut microbiota, including *Lactobacillus* and *Bacteroides* [32, 34], the richness and diversity of the mice/rat gut microbiota, and the ratio of *Firmicutes* to *Bacteroides* [37], the effects of the gut microbiota on the biological functions of these organs and the pathogenesis of various diseases remain to be revealed.

Here, we specifically investigated the role of the inflammatory activity to the development of psychological disorder depression and the effect of melatonin to the liver metabolic disorder in DSS treatment rats. We first determined that DSS exposure induces significant changes in behaviors. These findings indicate that DSS treatment result in unbalanced physiological processes; however, it is still uncovered but one possibility is LPS. Overrepresented gram-negative taxa were observed in depression/anxiety (DEP/ANX) patients [73]; meanwhile, phylogenetic investigation of communities by reconstruction of unobserved states (PICRUSt) and plasma analyses showed that LPS biosynthesis genes and LPS were overrepresented in the gut microbiome and plasma of DEP/ANX subjects [73], respectively. Interestingly, PICRUSt analyses indicate that LPS biosynthesis and LPS biosynthesis proteins markedly increased in DSS rats, which result in LPS significantly increased plasma. In addition, astrocytes and microglia in the hippocampus were activated induced by LPS invasion [84, 85]. Subsequently, inflammation-related cytokines, such as TNF-*α* and IL-1*β*, were increased in the hippocampus as well as the nuclear factor kappa-B (NF-*κ*B) pathway [31, 84, 85]. How LPS invaded the brain? The possibility is the collapse of the blood brain barrier (BBB). The increased expression of the gut and BBB permeability marker zonulin was observed in rats treated with DSS. Previous research suggested that elevated plasma zonulin strongly reflects increased gut and BBB permeability [73, 86, 87]. BBB permeability is regulated by both gut and neuroinflammation and is often used as an indicator of neuroinflammation [88–90]. In this study, supplementation with melatonin markedly improved gut leak, but there is no positive effect in the reduction of LPS, TNF-*α*, and IL-1*β*. As we all know, the ratio of *Firmicutes* to *Bacteroidetes* is considered to be a key index for the state of gut microbiota [69, 70]. Although supplementation with melatonin had a slight effect on the relative abundance of *Firmicutes* and *Bacteroidetes*, the ratio of *F*/*B* decreased significantly, which means the melatonin improved the structure of gut microbiota, since *Lactobacillus rhamnosus*, *Lactobacillus acidophilus*, and *Lactobacillus reuteri*, which belonged to *Bacilli*, have been considered to have anti-inflammatory function [91–93], whereas higher *Clostridia* abundance is usually associated with gut inflammation [94–96]. *Lactobacillus reuteri* has a beneficial emotional effect, while *Lactobacillus* can provide an increased protective mechanism against the adverse effects of *Clostridia*, or may occupy a niche originally occupied by *Clostridia* or *Actinobacteria* [71]. Notably, when we analyzed the relative abundance of *Bacilli* and *Clostridia*, we found that supplementation with melatonin significantly increased *Bacilli* and decreased *Clostridia*, respectively. Oral administration of melatonin in high-fat diet- (HFD-) fed mice markedly decreased the abundance of *Lactobacillus* [34], whereas oral administration of melatonin in healthy mice increased the abundance of *Lactobacillus* [32]. There are five genera (reduction of *Streptococcus* and *Enterococcus* and enrichment of *Lactobacillus* and *Clostridium*) that markedly changed after DSS rat were treated with melatonin. Interestingly, the observed behavior, gut leak, and proinflammatory changes after melatonin supplementation could be regulated partially by SCFAs. Indeed, recent research has demonstrated that SCFA plays a key role in modulating microglia maturation, morphology, and function [97]. Surprisingly, transplant with SCFA-producer *Clostridium tyrobutyricum* or *B. thetaiotaomicron* or supplementation with SCFAs could restore BBB integrity [98]. In the present study, *Lactobacillus* and *Clostridium* changed significantly when supplementation with melatonin and both of them belong to SCFA-producing genera. These data suggest that gut microbiota is a complex community and parts of probiotics are hardly demonstrating the signature of depression. However, a common signature of these relative studies was enhancement of gut inflammation in depression patients or rodent animals [99, 100] and lower permeability in antidepressant controls [73, 101, 102]. This suggests that metabolites derived from the gut microbiota may continuously affect the physiological state of the BBB. However, it is unclear whether other SCFAs or microbiota metabolites, even microbial species, may affect the permeability of BBB. This finding is also significant in other physiological processes, indicating that metabolites that do not normally enter the brain may cross the BBB depending on the state of the microbiome.

Research on depression usually focuses on the central and peripheral nervous system, not the liver. Our previous research showed that depression induced by chronic unpredictable mild stress in rats changed liver metabolism [28]. Also, germ-free mice that underwent fecal microbiota transplantation from major depressive disorder patients showed metabolic disorder [27]. Based on the previous study, we focused on the relationship of melatonin supplementation and FXR-FGF 15 and ASK1 signaling pathways, which are correlated to liver metabolism [23, 35]. In this section, we found that melatonin inhibited the overactivation of FXR-FGF 15, ASK1 signaling pathways, and its downstream cascade-p38 in a DSS-induced depression rat model. A previous study has demonstrated that melatonin safeguards against fatty liver by antagonizing TNF receptor-associated factor- (TRAF-) mediated ASK1 deubiquitination and stabilization in a *β*-arrestin-1-dependent manner [35]. Huang et al. reported that theabrownin from Pu-erh tea attenuates hypercholesterolemia via modulation of gut microbiota and bile acid metabolism [23]. Interestingly, in the present research, supplementation with melatonin suppressing microbes (*Streptococcus* and *Enterococcus*) is relevant to BSH activity [23]. Additionally, administration with melatonin inhibited the overactivation of the FXR-FGF 15 signaling pathway. Inhibited activation of FXR in intestinal results in decreased production of FGF15/FGF19 along with subsequent reduced FGF15/FGF19-FGFR4 signaling coupled with reduced cholesterol levels in the liver and plasma [23]. Melatonin significantly inhibits the expression levels of ASK1 and p-ASK1 and its downstream p38 and p-p38 pathways. As a mitogen-activated protein kinase (MAPKKK), ASK1 initiates and maintains p38 activation to induce apoptosis. Since ASK1 is overactivated in DSS-induced liver metabolic disorders, ASK1 can be an ideal drug target for treating liver metabolism disorders [103–105]. According to our findings, melatonin inhibits the activation of the ASK1 pathway and its downstream p38 may be a promising strategy to alleviate liver metabolism disorders. In combined previous studies, we propose that long-term administration of DSS or HFD may induce depression and metabolic disorder [23, 34, 35, 106]. The use of melatonin in DSS- or HFD-induced depression may not only alleviate the behavior but also improve disordered metabolism. This speculation needs further research to confirm.

In conclusion, our results suggest that (1) treatment with DSS induced depression-like behavior and neuroinflammation in rats via alteration of gut microbiota, (2) supplementation with melatonin reversed depression-like behavior and neuroinflammation via increased SCFA producer and enhanced the integrity of BBB, and (3) administration with melatonin decreased the activation of FXR-FGF15 and ASK1 signaling pathways and contributed to improved metabolic disorder. These results support the therapeutic value of melatonin in DSS-induced depression and/or liver metabolic disorder in the clinic.

## Data Availability

All data needed to evaluate the conclusions in the paper are present in the paper and/or Supplementary Materials. Additional data related to this paper may be requested from the authors.

## Supplementary Materials

Supplementary materials Fig. S1: PICRUSt analyses predicted that rats treated with DSS produce more LPS and supplementation with melatonin had no effect to improve this change. ^∗^*p* < 0.05.
