# Supplementary material for: Melatonin Alleviates Neuroinflammation and Metabolic Disorder in DSS-Induced Depression Rats
Source: Oxid Med Cell Longev. 2020 Jul 30;2020:1241894. doi: 10.1155/2020/1241894 (PMC7415091; doi:10.1155/2020/1241894)
Supplement: Supplementary materials — Fig. S1: PICRUSt analyses predicted that rats treated with DSS produce more LPS and supplementation with melatonin had no effect to improve this change. ∗p < 0.05. [file 1241894.f1.docx]

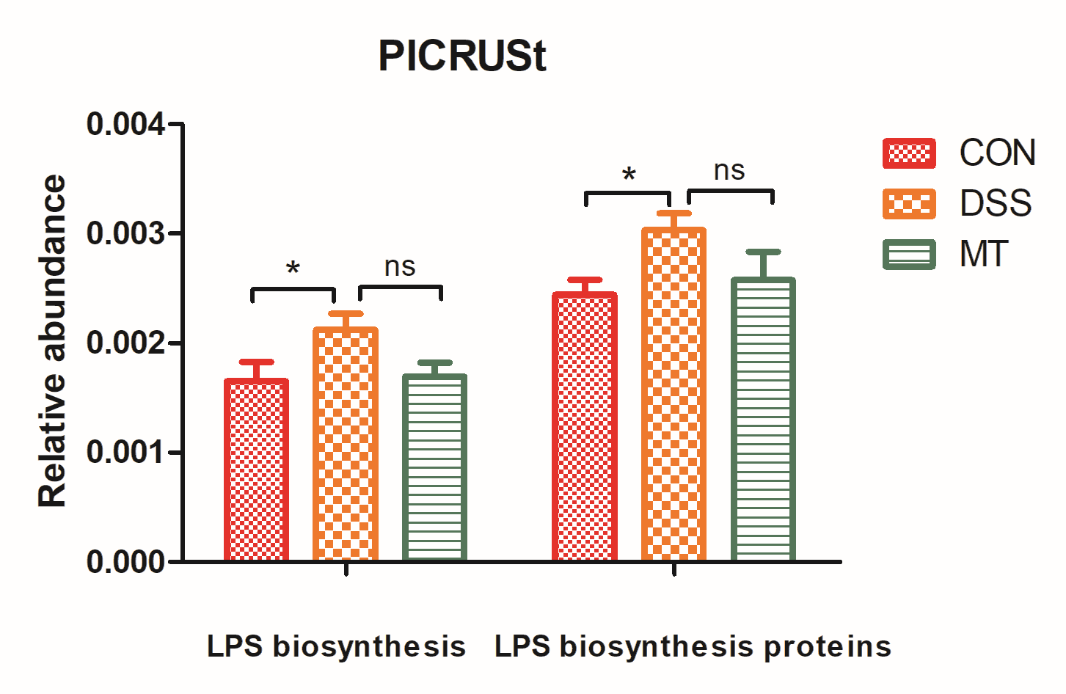


Fig. S1. PICRUSt analyses predicted that rats treated with DSS produce more LPS and supplementation with melatonin had no effect to improve this change. **p*<0.05
